# Translation equations to compare ActiGraph GT3X and Actical accelerometers activity counts

**DOI:** 10.1186/1471-2288-12-54

**Published:** 2012-04-20

**Authors:** Leon Straker, Amity Campbell

**Affiliations:** 1 School of Physiotherapy and Curtin Health Innovation Research Institute, Curtin University, Perth, Australia; 2 School of Physiotherapy, Curtin University, Perth, WA 6845, Australia

**Keywords:** Accelerometry, Actical, ActiGraph, Translation equations

## Abstract

**Background:**

This study aimed to develop a translation equation to enable comparison between Actical and ActiGraph GT3X accelerometer counts recorded minute by minute.

**Methods:**

Five males and five females of variable height, weight, body mass index and age participated in this investigation. Participants simultaneously wore an Actical and an ActiGraph accelerometer for two days. Conversion algorithms and R^2^ were calculated day by day for each subject between the omnidirectional Actical and three different ActiGraph (three-dimensional) outputs: 1) vertical direction, 2) combined vector, and 3) a custom vector. Three conversion algorithms suitable for minute/minute conversions were then calculated from the full data set.

**Results:**

The vertical ActiGraph activity counts demonstrated the closest relationship with the Actical, with consistent moderate to strong conversions using the algorithm: y = 0.905x, in the day by day data (R^2^ range: 0.514 to 0.989 and average: 0.822) and full data set (R^2^ = 0.865).

**Conclusions:**

The Actical is most sensitive to accelerations in the vertical direction, and does not closely correlate with three-dimensional ActiGraph output. Minute by minute conversions between the Actical and ActiGraph vertical component can be confidently performed between data sets and might allow further synthesis of information between studies.

## Background

Insufficient moderate-vigorous physical activity and too much sedentary behaviour are physical activity behaviours that are both recognised as significant public health issues [1,2]. A comprehensive body of research has attempted to capture physical activity behaviours to inform prevention policies, interventions and activity guidelines, as well as better understand the relationship with disease. Initially this research was hampered by reliance on self-report, which has been shown to be biased and inaccurate [3-5]. The development of activity monitors allowed an objective measure of intensity, duration and pattern of activity, which has led to a better understanding of the importance of physical activity behaviours [5,6]. For example, there is evidence that the relationship between physical activity behaviours and adiposity is strengthened when objective monitors are used rather than questionnaires in both children [7,8] and adults [4,9]. Similarly, it has been demonstrated that using activity monitors rather than questionnaires increases the likelihood of associations being detected between physical activity and a variety of health outcomes [10].

Accelerometers have become the most accurate, feasible and widely used available activity monitor device [6,11]. Studies using a number of different accelerometers have been reported [12-17], with researchers required to weigh the differing costs, unit dimensions, technical specifications, outputs and evidence for reliability and validity when deciding which model to utilise. Given the number of available devices, it is unlikely that one device will become universally adopted [18]. Therefore comparisons/standards that apply across the various units are necessary, a notion that is becoming increasingly recognised [19].

Two devices appear to be the most frequently used; the Actical (Mini Mitter Co., Inc,. Bend OR) and the ActiGraph (ActiGraph, LLC, Fort Walton Beach, FL). Historically, the single plane (vertical acceleration only) ActiGraph (ACG: model 7164) was the most frequently utilised device in research and represented a major technical advancement in being much smaller (51 × 41 × 15 mm; 43 g) than previous devices [20]. This popularity has continued, with the single plane ActiGraph utilised to collect the largest accelerometer data set recorded to date as part of the National Health and Nutritional Examination Survey (NHANES) in the US [21,22]. The Actical accelerometer is a newer and smaller accelerometer (28 × 27 × 10 mm: 17 g) that has become widely used [2,23] with the advantage of being ‘omni-directional’ [24]. Whilst the specific sensitivities in resultant three-dimensions have never been published, the combined three-dimensional output provides a theoretically more comprehensive assessment of body movements and has demonstrated higher correlations with energy expenditure in adults [25] and children [26]. Comparisons of reliability and validity typically find that the multiple axis models report marginally higher validity than single plane models [23,27]. Perhaps in response to both the Actical and validity evidence, the ActiGraph has recently evolved to offer acceleration outputs in each of the three planes of movement (ACG model: GT3X ) as well as the combined three-dimensional output, in a more streamlined model (dimensions: 38 × 37 × 18 mm and weight 27 g).

Methodological advancements regarding accelerometers to date have focused on validating the energy expenditure estimated by accelerometers [23,28]. However, there is growing awareness of the importance of activity in addition to energy expenditure and many research studies are now focusing on accelerometer counts [29-33]. Attempts to capture different aspects of the pattern of activity have used count thresholds, for example to define breaks in sedentary behaviour [8,32,34] or to determine total time spent performing moderate to vigorous physical activity [8].In order to facilitate comparisons of categorised physical activity between studies attempts to quantify standard cut offs have been performed [35-37].

Given the importance of the outputs from objective measures and the popularity of both the Actical and ActiGraph devices in contemporary literature, the ability to compare information between devices has been recognised as important [5,18,19]. This includes both comparisons between reported daily data as well as the accurate determination of equivalency of thresholds for activity categories between devices. However, this synthesis of results is currently not possible, given that the Actical and ActiGraph counts are not directly comparable [18] as they are arbitrary units (counts per minute) from technically different devices. The raw data from activity monitors is converted into an activity count over a user defined interval following some level of filtering. Therefore, the magnitude of the counts depends on the different electrical and/or mechanical characteristics of the activity monitor, along with the level of filtering, resulting in variation between brands [18,29,38]. This issue was recently addressed and a conversion algorithm for daily recordings was developed between the single plane ActiGraph and the Actical [18]. However due to a phase shift in their data that resulted in increasing discrepancies between the minute by minute conversions they were only able to provide a comparison for an average total daily accelerometer count [18]. While this is useful, it does not allow for conversions of shorter time periods which is particularly relevant, given the recognised importance of shorter bouts of activity and within day variability [30,31,33,39]. Further data is therefore required to determine whether a minute by minute conversion is feasible and to establish a conversion algorithm between the tri-axial ActiGraph and the Actical. This minute by minute conversion could also be utilised to verify equivalent activity thresholds between devices, which is critical for physical activity categorisation. Therefore, this study aimed to develop a translation equation to enable comparison between Actical and ActiGraph accelerometer counts recorded minute by minute.

## Methods

The protocol for this study was approved by the Curtin University Human Ethics Review Committee. Ten healthy adults (5 males and 5 females) provided informed consent and participated in this study. The participants were a convenience sample selected to represent both sexes and a range of ages (mean 37.5, range 26–52), heights (mean 174.5 cm, range 158–195), weights (mean 72.1 kg, range 54–104) and daily activity patterns (mainly sedentary work, mainly active work, no leisure physical activity, regular moderate/vigorous leisure activity).

Participants were provided with one Actical and ActiGraph fixed directly next to each other on an adjustable belt. Participants were instructed on the accurate positioning of the accelerometers; worn securely over the right anterior superior iliac crest, and asked to wear the monitors for two days. Participants were also required to complete a simple activity diary, where any changes to activity/unusual activities and times of any device removal/re-application were recorded.

Three different devices of each model were utilised due to the differences that have been demonstrated to exist between units. Prior to being worn by the participants, the devices were calibrated and configured for recording using the same computer. This ensured the internal clocks were configured to precisely the same time in order to minimise phase shift between units.

### Data processing and statistical analyses

Each participant’s counts per minute (cpm) data was output and graphed for visual inspection using Microsoft Excel (Microsoft Corporation inc.). Visual inspection in combination with diary information were utilised to both remove sleep and non-wear time and examine for phase shift. One participant’s data was repeated following the detection of phase shift. The source of this phase shift was not resolved; however the problem did not occur in this participant’s second set of data.

Scattergrams and regression equations were then calculated for each day of each participant’s data using Microsoft Excel. Linear regression equations were fitted with the intercept set to zero. Three different comparisons were made: 1) between the ActiGraph X (vertical axis) component and Actical, 2) between the ActiGraph vector output and Actical, and 3) between a custom calculated vector (ActiGraph custom vector) and the Actical. Finally, all the collected data (every day/participant) were combined and overall scattergrams and regression equations were calculated and a Bland-Altman plot prepared for comparison 1).

## Results

Participants wore the accelerometers for an average of 13 hours and 48 minutes each day (standard deviation 2 hours and 30 minutes). The results of the regression equation and R^2^ for each participant/day (Table 1) suggest a stronger relationship between the ActiGraph X axis and Actical activity cpm (R^2^ range: 0.514 to 0.989 and average: 0.822) than both the ActiGraph vector (R^2^ range: 0.002 to 0.930 and average: 0.404) and the custom vector (R^2^ range: 0.241 to 0.931 and average: 0.638).

**Table 1:** Relationships between three ActiGraph counts per minute outputs and Actical counts per minute output, for each subject each day

	**ActiGraph X axis and Actical**				**ActiGraph vector and Actical**				**ActiGraph custom vector and Actical**			
	**Day 1**		**Day 2**		**Day 1**		**Day 2**		**Day 1**		**Day 2**	
	**Regression Equation**	**R**^**2**^	**Regression Equation**	**R**^**2**^	**Regression Equation**	**R**^**2**^	**Regression Equation**	**R**^**2**^	**Regression Equation**	**R**^**2**^	**Regression Equation**	**R**^**2**^
Subject 1	y = 1.250x	0.868	y = 1.250x	0.884	y = 15.878x	0.383	y = 15.951x	0.490	y = 1.619x	0.806	y = 0.943x	0.791
Subject 2	y = 1.214x	0.585	y = 1.214x	0.514	y = 36.523x	0.194	y = 32.16x	0.190	y = 2.022x	0.305	y = 1.529x	0.249
Subject 3	y = 1.506x	0.641	y = 1.506x	0.797	y = 60.46x	0.561	y = 35.488x	0.109	y = 2.513x	0.516	y = 1.98x	0.544
Subject 4	y = 1.267x	0.777	y = 1.267x	0.850	y = 31.594x	0.251	y = 51.379x	0.771	y = 1.788x	0.517	y = 1.784x	0.772
Subject 5	y = 1.174x	0.838	y = 1.145x	0.935	y = 54.667x	0.408	y = 38.325x	0.241	y = 1.953x	0.580	y = 2.112x	0.684
Subject 6	y = 1.281x	0.813	y = 1.232x	0.954	y = 31.494x	0.398	y = 26.574x	0.564	y = 1.833x	0.653	y = 1.614x	0.858
Subject 7	y = 1.139x	0.855	y = 1.117x	0.793	y = 36.731x	0.242	y = 32.263x	0.054	y = 1.188x	0.508	y = 1.852x	0.241
Subject 8	y = 0.804x	0.981	y = 0.793x	0.966	y = 21.608x	0.841	y = 20.647x	0.687	y = 0.925x	0.915	y = 0.896x	0.833
Subject 9	y = 1.145x	0.947	y = 1.106x	0.989	y = 5.752x	0.930	y = 4.727x	0.002	y = 1.392x	0.892	y = 1.330x	0.931
Subject10	y = 1.095x	0.827	y = 0.844x	0.622	y = 19.354x	0.527	y = 17.427x	0.244	y = 1.316x	0.609	y = 1.198x	0.563

**Figure 1:**
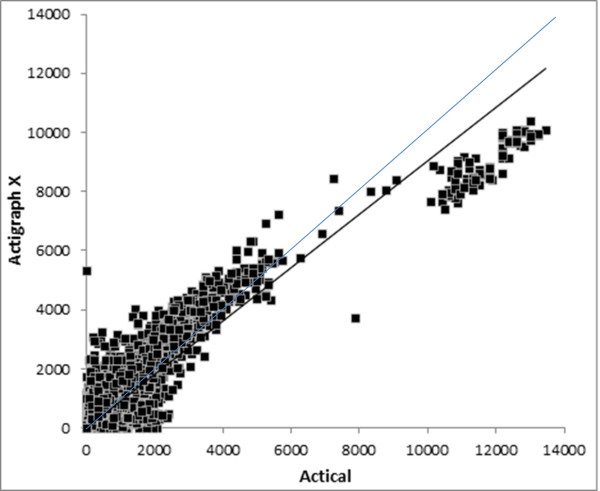
**Scattergram of ActiGraph vertical component and Actical cpm.** Dark line shows regression line: y = 0.905x with R^2^ = 0.865, faint line shows line of identity.

**Figure 2:**
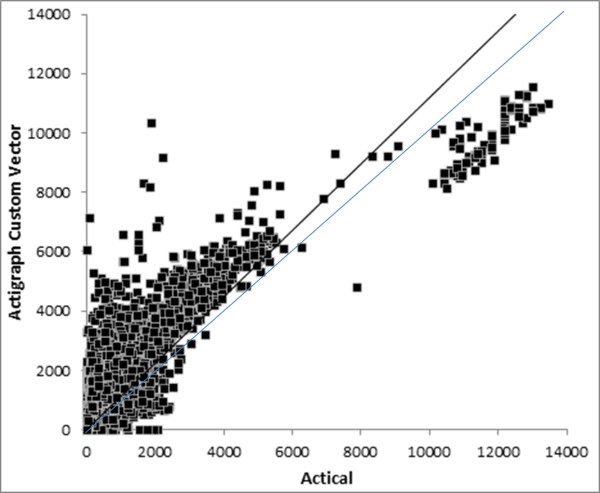
**Scattergram of ActiGraph custom vector and Actical cpm.** Dark line shows regression line : y = 1.229x with R^2^ = 0.635, faint line shows line of identity.

**Figure 3:**
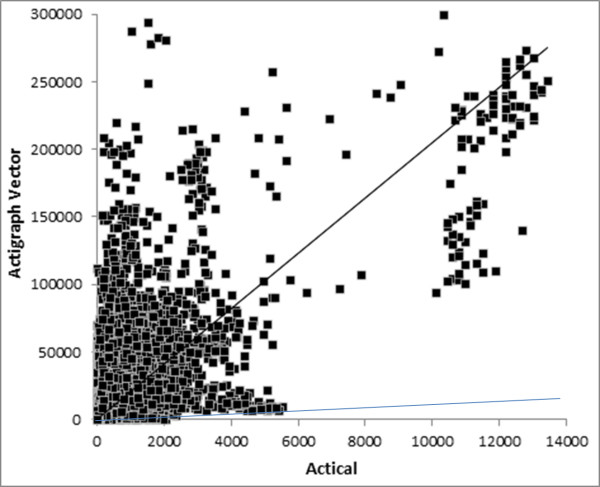
**Scattergram of ActiGraph vector and Actical cpm.** Dark line shows regression line: y = 20.493x with R^2^ = 0.382(note changed Y axis scale) , faint line shows line of identity.

**Figure 4:**
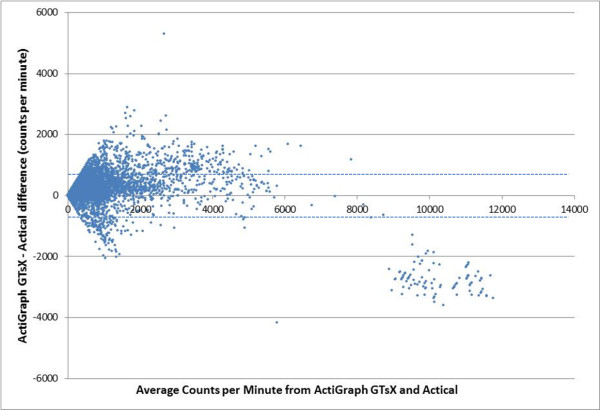
Bland-Altman plot showing differences between ActiGraph vertical component and Actical with 95 % confidence interval.

The overall R^2^ and scatter graphs (Figures 1, 2, 3) for the three sets of comparisons further support a stronger relationship between the ActiGraph vertical component and the Actical (R^2^ = 0.865) than the ActiGraph vector (R^2^ = 0.382), and ActiGraph custom vector (R^2^ = 0.635). The scattergram for the relationship between the ActiGraph vector and Actical (Figure 3) suggests that the ActiGraph vector overestimates activity count magnitude in comparison to the Actical. The count mean and standard deviation across all days for all subjects for the Actical was lowest (293.7 ±977.4), followed by the ActiGraph vertical component (377.5 ±977.4). The ActiGraph custom vector (694.1 ±1261) and the ActiGraph vector (12892 ±29401) appeared to overestimate activity compared to the Actical. Figure 4 shows a Bland-Altman plot with 95 % confidence intervals (+ 713 cpm).

## Discussion

Translating activity data (cpm) collected with different devices has recently been recognised as an important physical activity research issue [18,19,39]. This study outlines the first ActiGraph vs Actical activity count translation equations for within day activity.

The results of this study indicate a consistent, strong relationship between the ActiGraph vertical component and the Actical counts per minute, as indicated by the high day by day and overall squared correlations. Eslinger and Tremblay [38] compared average counts per minute over 7 minute trials between single plane ActiGraphs and Acticals following the mechanical generation of known accelerations. We utilised their reported data, albeit from only 7 data points, to calculate a comparable regression equation (y = 0.922x, R^2^ 0.824). The similarity between these two equations developed from very different sets of data (mechanical vs biological) supports the validity of the equations reported here. The only prior study comparing biological acceleration between single plane ActiGraphs and Acticals has limited application as it only allowed data conversions between total daily recordings (y = 38.5 + 0.947x) and was limited to the original uniaxial model of the ActiGraph (Paul et al 2007).

The relationship between the ActiGraph vector and the Actical was the weakest overall and may have been affected by the overestimation of the contribution of movement in the z axis; with the vector calculation used by ActiGraph *cubing* the z component [√(X^2^ + Y^2^ + Z^3^)]. Therefore, the custom ActiGraph vector calculation we developed used the more traditional ‘resultant vector’ calculation [√(X^2^ + Y^2^ + Z^2^)], and while this did result in a stronger relationship with the Actical, it was still weaker than the ActiGraph vertical relationship. This supports previous research suggesting the Actical is most sensitive to movements in the vertical plane [13].

The scattergrams between the activity monitors suggest a largely linear relationship between the Actical and the ActiGraph vertical component (Figure 1) and custom vector (Figure 2). However, the ActiGraph appears to be more sensitive to activity below around 8,000 cpm, whilst the Actical appears to be more sensitive to activity above 8,000 cpm. Interestingly, the Actical/ActiGraph vertical scattergram (Figure 1) appears to include very few outliers, whereas Actical/Actigraph custom vector scattergram (Figure 2) shows considerably more outlier data points where either the ActiGraph over-estimated or the Actical under-estimated activity. This also suggests the Actical is less sensitive to movements in the non-vertical planes.

It has been suggested that the most meaningful contribution of activity monitors is the recording of intermittent, light-intensity activities such as walking and incidental physical activity which are typically less memorable and likely associated with inaccuracies when using self-report measures [30,39]. This requires the establishment of suitable count thresholds in order to categorise activity. For example, 100 counts per minute is a common threshold used for sedentary behaviour in papers that utilised the uniaxial ActiGraph [31,33,40]. Similarly, Wong et al [37] recommended 100 counts per minute as a threshold for sedentary behaviour using the Actical device. Whilst these two values are numerically the same, they do not represent the same amount of activity due to the abovementioned, known differences between devices [18,29,38]. However, using the regression equation presented here, thresholds can now be accurately established between devices. For example 100 counts recorded with the ActiGraph vertical component is equivalent to 91 counts recorded with the Actical. Alternatively, 100 counts recorded with the Actical is equivalent to 110 counts recorded with the ActiGraph vertical component, or 81 counts recorded from the ActiGraph custom vector. Given earlier and broader use of the ActiGraph, we recommend Actical data be processed with activity thresholds translated to match ActiGraph thresholds. However the sensitivity of accelerometer results to corrections based on the equations reported here are likely to be fairly small given the sensitivity and specificity evidence around a sedentary threshold reported by Wong et al [37].

A limitation of the current study was the small amount of data collected in the 6,000-8,000 cpm range and the use of a linear equation. However, the inclusion of a range of people in regards to gender, age, height, weight and activity levels and collection of data over a wide range of free living occupational and non-occupational activities rather than just treadmill or other laboratory tasks can be considered strengths of this study and highlights the applicability of the presented equations.

## Conclusions

The regression equations developed in this investigation allow the synthesis of data and activity thresholds between studies utilising the Actical and ActiGraph GT3X activity monitor devices. Given the popularity of these two models for the acquisition of large data sets such as: 2503 New Zealanders (ActiGraph), [41], 6329 Americans (ActiGraph) [42] and, 1608 Canadians (Actical) [2], the ability to compare between studies should facilitate a more comprehensive understanding of the relationship between physical activity and disease.

## Competing of interest

There were no competing interests associated with this study.

## Authors’ contributions

AC completed the ethics application, data collection, data quality inspection and analysis. LS designed this project, assisted with data collection quality inspection and analysis. Both authors were involved with results interpretation and manuscript preparation. Both authors read and approved the final manuscript.

## Pre-publication history

The pre-publication history for this paper can be accessed here:

http://www.biomedcentral.com/1471-2288/12/54/prepub
